# Combining antiviral drugs with BET inhibitors is beneficial in combatting SARS‐CoV‐2 infection

**DOI:** 10.1002/ctd2.66

**Published:** 2022-05-06

**Authors:** Arpan Acharya, Tatiana G. Kutateladze, Siddappa N. Byrareddy

**Affiliations:** ^1^ Department of Pharmacology and Experimental Neuroscience University of Nebraska Medical Center Omaha Nebraska USA; ^2^ Department of Pharmacology University of Colorado School of Medicine Aurora Colorado USA

**Keywords:** BET, inhibitor, PI3K, remdesivir, SARS‐CoV‐2, SF2523

## Abstract

The COVID‐19 pandemic caused by the novel coronavirus SARS‐CoV‐2 has resulted in more than 500 million cases and 6 million deaths. Several antiviral therapies and vaccines have been developed to mitigate the spread of this infection. However, new approaches are required to battle emerging SARS‐CoV‐2 variants containing mutations that can reduce the vaccines' efficacy. The use of a combination of antiviral drugs with inhibitors of mammalian target of rapamycin (mTOR) signalling pathways has emerged as one of the promising novel approach. In this study, we have shown that SF2523, a dual activity small molecule that inhibits PI3K and BRD4, acts synergistically with the antiviral drugs remdesivir (RDV) and MU‐UNMC‐2. Our findings suggest that the mTOR pathways are necessary for SARS‐CoV‐2 pathogenesis in human cells and that targeting PI3K/BET (bromodomain and extra‐terminal domain proteins) alone or combined with antiviral therapies is beneficial in mitigating SARS‐CoV‐2 and its variants of concern (VOCs).

## Graphical abstract

The mammalian target of rapamycin (mTOR) pathways are necessary for SARS‐CoV‐2 pathogenesis in human cells, and targeting PI3K/BET (bromodomain and extra‐terminal domain proteins) alone or combined with antiviral therapies is beneficial in mitigating SARS‐CoV‐2 and its variants of concern (VOCs).

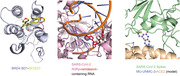

The SARS‐CoV‐2 coronavirus hijacks the host cell's molecular machinery to infect the cell, survive and replicate. The pathophysiology of COVID‐19 follows a biphasic pattern. The initial acute phase of infection manifests in viral infection‐driven symptoms, including sore throat, fever, fatigue, dry cough, and diarrhoea.^1^, ^2^ In severely ill patients, this is followed by an immunopathologic phase that includes the development of acute respiratory distress syndrome (ARDS), systemic inflammation, and cytokine storm, which is responsible for multiple organ failures and a higher rate of fatalities.^3^ Patients with certain comorbid conditions, such as hypertension, cardiovascular disease, diabetes, and chronic obstructive pulmonary disorders (COPD), also have a higher probability of developing ARDS. Moreover, a subpopulation of patients develops neurological disease manifestations such as loss of taste and smell, dizziness, confusion, ataxia, seizures, and Guillain–Barré syndrome (GBS).^4^ SARS‐CoV‐2 infection may lead to hyperactivation of mammalian target of rapamycin complex 1 (mTOR1). This results in the production of inflammatory cytokines and the survival of infected cells.

A recent study by Gordon et al.^5^ used a proteomic approach to explore the interactions of SARS‐CoV‐2 proteins with cellular targets in human cells. They identified 67 potential interactions between human proteins and viral proteins essential for the SARS‐CoV‐2 lifecycle and demonstrated an interaction of bromodomain and extra‐terminal domain proteins 2 and 4 (BRD2/BRD4) with the E protein of SARS‐CoV‐2. Another study that utilised CRISPRi screening showed that BRD2 inhibition downregulates angiotensin converting enzyme 2 (ACE2) expression and controls the hyperactive immune response in COVID‐19 patients by downregulating interferon‐stimulated genes (ISGs).^6^ Further, Gilham et al. reported that apabetalone (RVX‐208), a bromodomain and extra‐terminal domain family of proteins (BET) inhibitor, blocks SARS‐CoV‐2 infection through a reduction in ACE2 expression,^7^ and Qiao et al. showed that transcriptional repression of androgen receptors using BET inhibitors also leads to a reduction in SARS‐CoV‐2 infection.^8^ Furthermore, the cytokine storm associated with COVID‐19 leads to cardiomyocytes, and BET inhibitors may protect COVID‐19 patients from cardiomyocytes.^9^ Together, these studies indicate that BET proteins can be potential targets for developing therapeutics against SARS‐CoV‐2.^10^

The PI3K/Akt/mTOR pathway has been linked to viral infections.^11^ It has been reported that SARS‐CoV‐2 dysregulates the PI3K/Akt/mTOR pathway within the host cell to increase its survival and replication.^12^ La ribonucleoprotein 1 (LARP1), a major effector of the mTOR pathway, interacts with nucleocapsid protein (N).^13^ LARP1 downregulation by mTOR inhibitors blocks MERS virus replication and has an immunosuppressive function.^14^ We have shown that BRD2/BRD4 and mTOR are critical host factors responsible for the pathogenesis of SARS‐CoV‐2 based on the activity of the small molecule SF2523.^15^ SF2523 is a potent inhibitor of PI3Kα (IC_50_ = 34 nM), PI3Kγ (IC_50_ = 158 nM), DNA‐PK (IC_50_ = 9 nM), BRD4 (IC_50_ = 241 nM), and mTOR (IC_50_ = 280 nM). In in vitro cell culture models, SF2523 lowers the protein expression levels of MYCN and cyclin and inhibits the activation of AKT by blocking the phosphorylation of Ser473. In the in vivo mice model, SF2523 reduces the tumour volume without significant cytotoxicity to the treated animals.^16^ Collectively, these reports suggest a set of host machinery elements essential in viral pathogenesis that represent attractive targets for anti‐SARS‐CoV‐2 therapeutic intervention.

Considering the emergence of mutant variants and variants of concern (VOCs) of SARS‐CoV‐2, such as Delta (B.1.617.2) from India, Gamma (P1) from Brazil, Beta (B.1.351) and Omicron (B.1.1.529) from South Africa, and Alpha (B.1.1.7) from the UK^17^ with an attenuated response to vaccine candidates, we and others suggest a combinatorial approach may be better choice. This approach involves the use of a multiaction small molecule that inhibits multiple interactions necessary for viral pathogenesis in combination with other antiviral agents. Recently, we showed in vitro efficacy of SF2523 as a monotherapy and combined with remdesivir (RDV) or the newly developed inhibitor MU‐UNMC‐2. We found that SF2523, an inhibitor of PI3K‐α/mTOR/BRD4, effectively blocks the replication of SARS‐CoV‐2 and its VOCs, including delta and omicron.^15^ Furthermore, SF2523 acts in synergy with the antiviral drug RDV and MU‐UNMC‐2, a small molecule inhibitor that blocks the entry of SARS‐CoV‐2.^18^ We note that SF2523, RDV, and MU‐UNMC‐2 differ in their mechanisms of action (Figure 1). While SF2523 targets bromodomains of BRD4 and the catalytic domain of PI3K^16^ RDV, a nucleoside analog, incorporated into the SARS‐CoV‐2 RNA‐dependent RNA polymerase complex blocks RNA translocation and therefore inhibits SARS‐CoV‐2 replication.^19^, ^20^ MU‐UNMC‐2 was designed to disrupt the association between the SARS‐CoV‐2 spike receptor‐binding domain (RBD) and the human protein ACE2.^18^ The combinatorial approach increases the efficacy of the treatment, reduces the dosage requirements for the individual drugs, lowers the overall toxicity profile, and minimises the chances of developing drug resistance. We found that low doses compared to the corresponding IC_50_ values of the individual compounds (SF2523 and MU‐UNMC‐2) acted in synergy against SARS‐CoV‐2, thereby underscoring the benefit of the combinatorial approach. This novel efficient therapeutic approach to block SARS‐CoV‐2 infection and its emerging variants has significant potential and warrants more in‐depth structural and functional investigation.

**FIGURE 1:**
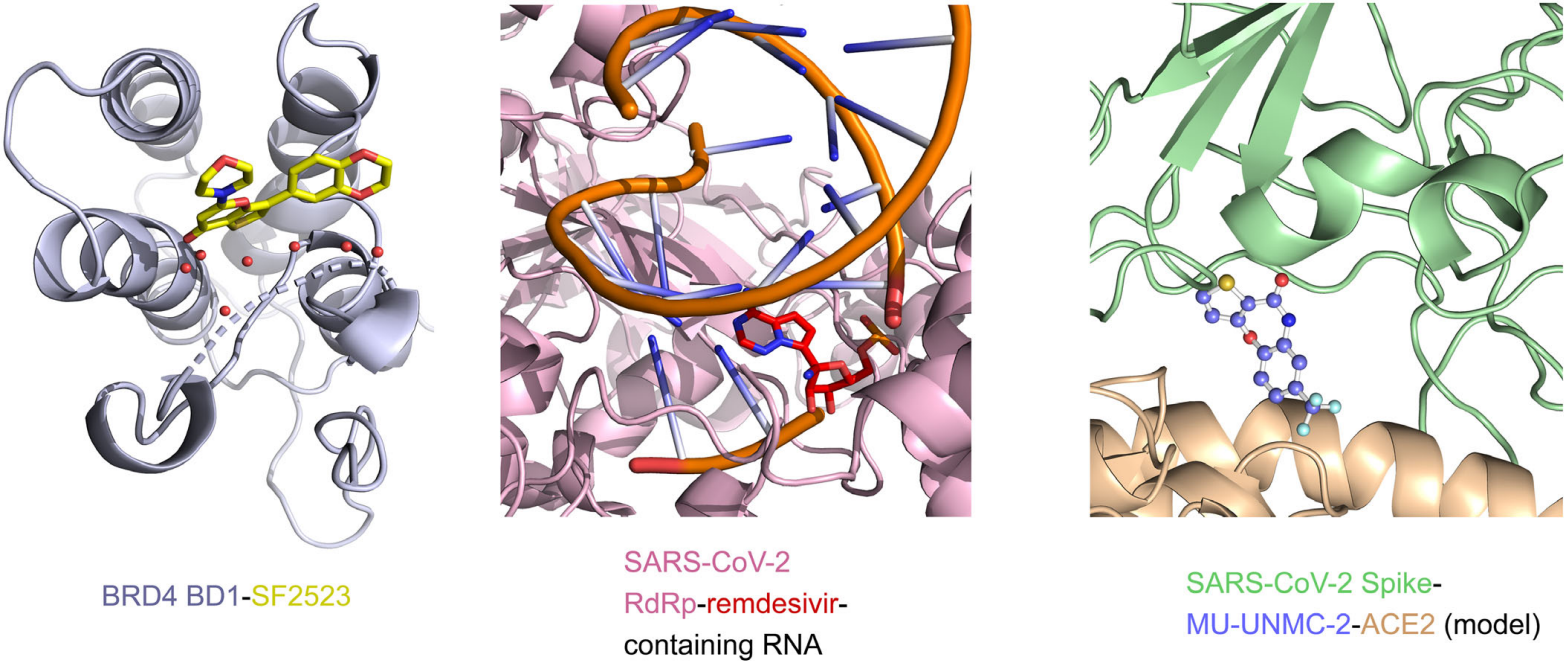
Structural mechanisms of inhibition by SF2523, remdesivir, and MU‐UNMC‐2. (Left panel) The crystal structure of BRD4 BD1 (light blue) in complex with SF2523 (yellow). Water molecules are shown as red spheres, PDB ID 5U28. (Middle panel) Cryo‐EM structure of SARS‐CoV‐2 RNA‐dependent RNA polymerase (pink) with remdesivir (red) incorporated at position ‐3 (structure 1), PDB ID 7B3B. (Right panel) A model of the ACE2(wheat)‐Spike(RBD)(green)‐MU‐UNMC‐2(blue) complex

## CONFLICT OF INTEREST

The authors declare no conflict of interest.
